# Nanoparticle-Based Paramagnetic Contrast Agents for Magnetic Resonance Imaging

**DOI:** 10.1155/2019/1845637

**Published:** 2019-05-05

**Authors:** Juan Pellico, Connor M. Ellis, Jason J. Davis

**Affiliations:** Department of Chemistry, University of Oxford, South Parks Road, Oxford OX1 3QZ, UK

## Abstract

Magnetic resonance imaging (MRI) is a noninvasive medical imaging modality that is routinely used in clinics, providing anatomical information with micron resolution, soft tissue contrast, and deep penetration. Exogenous contrast agents increase image contrast by shortening longitudinal (*T*_1_) and transversal (*T*_2_) relaxation times. Most of the *T*_1_ agents used in clinical MRI are based on paramagnetic lanthanide complexes (largely Gd-based). In moving to translatable formats of reduced toxicity, greater chemical stability, longer circulation times, higher contrast, more controlled functionalisation and additional imaging modalities, considerable effort has been applied to the development of nanoparticles bearing paramagnetic ions. This review summarises the most relevant examples in the synthesis and biomedical applications of paramagnetic nanoparticles as contrast agents for MRI and multimodal imaging. It includes the most recent developments in the field of production of agents with high relaxivities, which are key for effective contrast enhancement, exemplified through clinically relevant examples.

## 1. Introduction

Noninvasive imaging techniques support disease diagnosis and pathological characterisation with ease in a comparatively safe manner [1, 2]. These techniques include nuclear imaging modalities such as positron emission tomography (PET), single photon emission computed tomography (SPECT), computed tomography (CT), optical imaging (OI), ultrasound (US), and magnetic resonance imaging (MRI). All these present individual distinct advantages, as well as limitations which need to be considered within the aims of the specific study. MRI is routinely utilised because of its associated intrinsic high spatial resolution, deep tissue penetration, and three-dimensional anatomical information [3]. It is, thus, widely employed in clinics for the diagnosis and prognosis of a broad range of disease states. Despite this, MRI suffers from inherently low sensitivity; hence, exogenous contrast agents are applied to overcome this obstacle by shortening the relaxation times of bulk water [4]. Contrast agents not only enhance image contrast but also support multimodal imaging. They can be classified into two different groups depending on the operational mode. *T*_1_, also called positive contrast agents, shortens longitudinal relaxation times and brightens the accumulation area [5]. *T*_2_, or negative contrast agents, conversely shortens transversal relaxation times and darkens the immediate and surrounding area. From metal complexes to nanoparticles, different formulations have been employed as contrast agents for MRI with most of these based on the use of highly paramagnetic ions such as Gd^3+^, Mn^2+^, and Fe^3+^ [6]. They are usually utilised as coordination complexes with acyclic or cyclic chelate agents to reduce associated toxicity of the free metal ion. Although these molecular agents have been extensively used, paramagnetic metallic complexes have problems related to their fast excretion [7]. In terms of targeting and adding additional imaging modes, the use of low molecular weight lanthanide complexes is limited by high synthetic demand and the possibility of undesirable side reactions occurring during the synthesis. For these reasons, paramagnetic contrast agent research has focused on the development of nanoparticulate forms over the last few years [8, 9]. Paramagnetic nanoparticles present several advantages over traditional coordination complexes. Composition is readily tuneable as is size and shape. Magnetic characteristics are improved by geometric local density effects rendering markedly higher *T*_1_ and/or *T*_2_ relaxometric values than the corresponding coordination complexes. In addition, control over associated pharmacokinetics enables an increase in blood circulation time [10]. Ultimately, most nanoparticulate platforms are cleared by the reticuloendothelial system (RES) with the action of macrophages, such as Kupffer cells, generating uptake from the bloodstream to the liver and spleen. The rate of this process, known as opsonization, depends on the size of the nanoparticle and its chemical coating. Nanoparticles below 5–7 nm in diameter are able to pass through the kidney glomerulus triggering fast excretion through urine. Larger particles are excreted by the RES route with kinetics that can be tuned through the surface chemistry. The protein corona that develops on these particles during circulation is key to this and can be controlled by pegylation, for example, or a polymer coating such that blood circulation time is optimised [11]. This has a direct role in MRI acquisition, where a greater acquisition time increases signal-to-noise and consequently “image quality.” Another important feature within the use of nanoparticles is their high surface-to-volume ratio, supporting a high ligand (protein, antibody, and peptide) payload as well as tuneable and potentially accessible internal volume [12]. These features enable combination with drugs such that targeted, imaging-based, diagnosis may be followed by simultaneous therapy specific to that condition, known as theranostics [13].

Many different approaches have been used to develop paramagnetic nanoparticles for MRI, a few of which will be represented herein. In general, one of two synthetic strategies is followed: (1) formation of nanoparticles with the paramagnetic ion incorporated into the nanostructured framework and (2) postfunctionalisation of the particles with a lanthanide coordination complex. The first approach has been largely based on the synthesis of stoichiometric or nonstoichiometric nanoparticulate metal oxides such as Gd_2_O_3_, MnO, Mn_3_O_4_, Dy_2_O_3_, or *γ*-Fe_2_O_3_. This approach also includes NaGdF_4_, KGdF_4_, *β*-NaDyF_4_, NiFe_2_O_4_, and ZnFe_2_O_4_ nanoparticles as well as others [14–19]. The second strategy has been developed with a number of supporting nanoparticle scaffolds (silica, gold, micelles, polymers, and semiconducting quantum dots) which are subsequently doped with DTPA, DOTA, or derivatives [20–24]. This review focuses on the synthesis of Gd^3+^, Mn^2+^, Dy^3+^, and Ho^3+^ nanoparticles and their biomedical application as contrast agents for MRI and multimodal imaging.

## 2. Paramagnetic Ion-Based Relaxivity

The relaxivity value, in mM^−1^·s^−1^, quantifies the ability of a contrast agent to promote contrast in MRI. In paramagnetic ions, two different mechanisms should be considered, both an inner-sphere and an outer-sphere mechanism, respectively:

(1) $$\begin{array}{c} {\left(\frac{1}{T_{i}}\right)}_{\text{p}}={\left(\frac{1}{T_{i}}\right)}_{\text{IS}}+{\left(\frac{1}{T_{i}}\right)}_{\text{OS}},\;\;i=1,2. \end{array}$$

The inner-sphere mechanism is predominant in paramagnetic contrast agents. It represents the influence of the paramagnetic ion on highly local water protons and is sensitive to the chemical water exchange between the first coordination sphere (inner-sphere) of the paramagnetic ion and bulk water. In terms of longitudinal relaxation rate, a range of different factors are considered within the inner-sphere contribution as described in the following equation:

(2) $$\begin{array}{c} {\left(\frac{1}{T_{1}}\right)}_{\text{IS}}=P_{\text{m}}q\left[\frac{1}{\left(T_{1\text{m}}+\;\tau_{\text{M}}\right)}\right], \end{array}$$

where *P*_m_ refers to the mole fraction of the paramagnetic ion, *q* is the number of water molecules coordinated to the metal centre (hydration number), *T*_1m_ the relaxation time of the water protons bounded to the ion, and *τ*_M_ is the bound residence lifetime. By considering equation (2) and Solomon–Bloembergen–Morgan (SBM) theory, three important parameters must be taken into consideration in agent design: hydration number (*q*), residence lifetime (*τ*_M_), and the rotational correlation time (*τ*_R_) [25]. The hydration number is usually equal to 1 in kinetically stable lanthanide complexes though a broad range of chelate agents with larger hydration numbers (and higher longitudinal relaxivities) have also been reported [26, 27]. An increase in hydration can come with the cost of stability (either chemical or signal output) [4]. Residence lifetimes should scale inversely with strength of the magnetic field and optimal values have been found to be in the range of 1–30 ns [28]. In addition, it has been shown that *τ*_R_ governs the relaxivity when *τ*_M_ is in the optimal range. The best relaxometric performance is typically observed when *τ*_R_ is the range of a few nanoseconds [29].

Gadolinium (Gd^3+^) and manganese (Mn^2+^) are the most used paramagnetic *T*_1_ contrast agents for MRI. Gd^3+^ has 7 unpaired electrons in the 4f subshell and a high associated spin quantum number (*S* = 7/2). Mn^2+^ contains 5 unpaired electrons in its valence d orbitals and hence also a high spin quantum number (*S* = 5/2). Both of them present high magnetic moments, symmetric orbital ground states, large longitudinal electronic relaxation times (∼10^−8^ s), and fast water exchange kinetics [30, 31].

Although paramagnetic ions are more commonly used as *T*_1_ contrast agents, there are examples, such as Dy^3+^ and Ho^3+^, which present notable *T*_2_ contrast. In contrast to Mn and Gd, Dy^3+^ and Ho^3+^ have highly anisotropic ground states with substantial spin-orbit and Zeeman effects [32]. Therefore, these ions show very short electronic relaxation times (∼10^−13^ s) that are accompanied by high effective magnetic moment (*μ*_eff_ Dy^3+^ = 10.6 *μ*B). Due to these properties, Dy^3+^ and Ho^3+^ coordination complexes are found to affect primarily the transversal relaxivity (*T*_2_ contrast) with relaxivities increasing with magnetic field strength by a Curie relaxation mechanism. The latter points to facilitating an application of Dy^3+^/Ho^3+^ molecular complexes in ultrahigh field MRI [33, 34].

It has been reported that the inner-sphere contribution governs the transverse relaxation of bulk water in these complexes where slow water exchange results in better relaxivities [35, 36]. Very recently, it has been demonstrated that the incorporation of Dy-DOTA complexes into silica nanoparticles changes the relaxation mechanism towards that associated with an enhanced Curie outer-sphere contribution [20].

## 3. Paramagnetic Nanoparticles

A very broad range of nanoparticulate MRI contrast agents have been reported. This review will focus on a few of these (Table 1), namely, those associated with the paramagnetic ions Gd^3+^, Mn^2+^, Dy^3+^, and Ho^3+^, since they have been extensively studied and often show striking MRI performance.

### 3.1. Gadolinium

**Figure 1:**
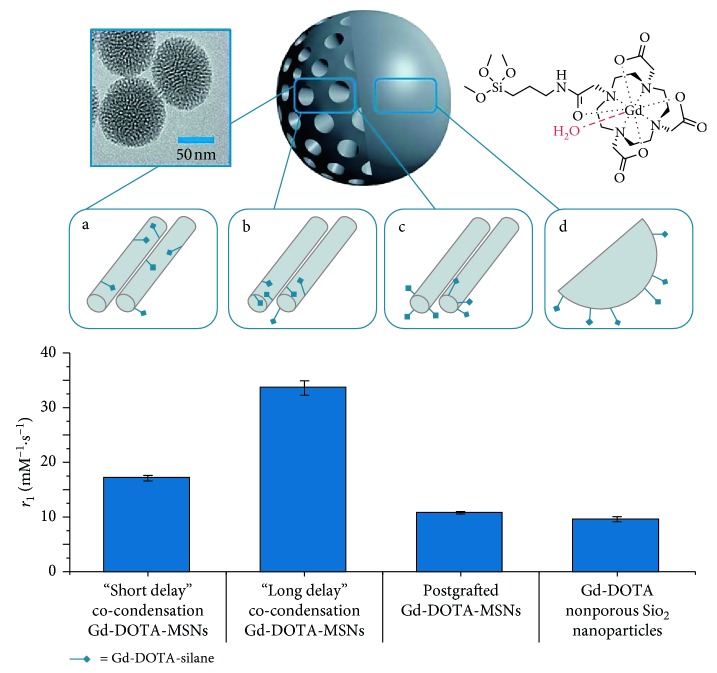
Typical transmission electron microscope image and schematic representation of Gd-DOTA-MSNs (66.3 ± 6.6 nm) prepared using (a) “short delay” co-condensation, where functionalities are internalised deeply in the structure (*r*_1_ = 17.14 ± 0.49 mM^−1^·s^−1^), (b) “long delay” co-condensation, where functionalities are internalised nearer to the porous openings (*r*_1_ = 33.57 ± 1.29 mM^−1^·s^−1^), and (c) postgrafting, where functionalities are loaded on external surfaces (*r*_1_ = 10.77 ± 0.22 mM^−1^·s^−1^). (d) Postgrafted Gd-DOTA-non-porous silica nanoparticles (*r*_1_ = 19.56 ± 0.47 mM^−1^·s^−1^) (reproduced from [71]).

A broad range of approaches have been applied to the incorporation of Gd chelates into nanoparticles, some of which are included here, since they enable high levels of chemical tailoring [70]. Of them, gadolinium-doped silica nanoparticles have been extensively reported. A study by Rieter et al., for example, utilised a luminescent [Ru(bpy)_3_]Cl_3_ core to which a coating of a silylated Gd complex was applied [37]. This produced stable particles with *r*_1_ values markedly greater than conventional molecular Gd chelates. This work was then taken further to produce mesoporous silica nanoparticles (MSNs) with even higher relaxivity values in an approach that has subsequently been adopted by many research groups [38]. Other work has demonstrated that the location of the Gd chelate within the MSNs greatly influences its relaxometric properties. The highest relaxivities have been specifically reported to occur when synthesis is by a long delay co-condensation process with *r*_1_ value of 33.6 ± 1.3 mM^−1^·s^−1^, higher than any previously reported Gd-DOTA silica nanoparticles and 20 times larger than free Gd-DOTA (Figure 1) [39]. These particles were then biotinylated showing that the large relaxivity of the particles was essentially unchanged on external biomodification but was then reversibly gateable on subsequent protein recognition [72].

Graphene oxide (GO) has also been used as a scaffold to integrate Gd-DOTA moieties. In one study, GO was first pegylated, functionalised with DOTA, and then metallated with Gd^3+^. These nanoparticles presented a large *r*_1_ value of 14.2 mM^−1^·s^−1^ measured at 11.7 T [41]. Another interesting scaffold for Gd-DOTA is the use of the tobacco mosaic virus (TMV). TMV is a rod-like plant virus formed by 2130 identical coat proteins assembled into a 300 × 18 nm hollow tube with a 4 nm pore channel. Gd-DOTA was loaded into the interior channel by a copper-catalysed azide-alkyne cycloaddition reaction yielding *r*_1_ = 10.9 mM^−1^·s^−1^. Surprisingly, this relaxivity increased to 29.7 mM^−1^·s^−1^ when the surface of the TMV was functionalised with silica [42].

In recent work, cerium oxide nanoparticles have been utilised to produce Gd-cerium nanoparticles with antioxidant capabilities. These nanoparticles, between 3 and 5 nm in size, rendered longitudinal relaxivities around 7–13 mM^−1^·s^−1^ [40]. Gd^3+^-impregnated nanodiamonds have also been used to produce CAs. These particles have been synthesised via an unusual detonation synthesis where an oxygen deficient trinitrotoluene/1,3,5-trinitroperhydro-1,3,5-triazine (TNT/RDX) mixture is detonated under an inert atmosphere. The nanodiamonds, formed in the early stages of the detonation under high pressure, reportedly are 4–5 nm in size with a narrow size distribution. These were then modified with Gd^3+^ at the surface carboxylic groups generating unprecedented transverse relaxivity (*r*_2_ = 332 mM^−1^·s^−1^) and corresponding *r*_1_ = 33.4 mM^−1^·s^−1^; the relaxivity mechanisms that are responsible for this high *r*_2_ value warrant further analysis [43]. Another interesting example is a report of the synthesis of protein-based nanocages. These were further functionalised with maleimide-DTPA-Gd^3+^ using a cysteine residue of the protein with the relaxometric properties analysed for four nanocages with different sizes (from 16.8 to 37.1 nm). The data revealed that higher *r*_1_ values (up to 47 mM^−1^·s^−1^ at 1.5 T) were obtained in larger nanocages due to the reduction in the tumbling rate of associated water molecules [44].

Gadolinium oxides are the most utilised alternatives to Gd chelates, where it has been found that decreasing particle diameter results in a progressive trend towards higher relaxivities. For instance, Park et al. showed that the highest relaxivities were obtained for nanoparticles synthesised with an average diameter of *d* = 1–2.5 nm [15]. It should be noted, however, that ultrasmall Gd_2_O_3_ have been found to form deposits in the brain and consequently there is a compromise between limiting the toxicity of the particles while maximising imaging potency. A study by Yin et al. produced silica nanoparticles coated in a Gd_2_O_3_ nanoshell of varying thicknesses. By systematically changing the thickness of the silica shell, the variations in relaxivity values could be investigated and demonstrated that a thinner shell resulted in larger *r*_1_ values [47].

**Figure 2:**
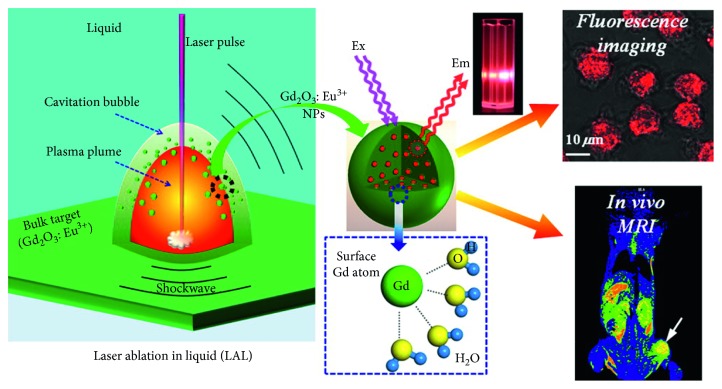
Figure illustrating the dual-modal imaging properties of Gd_2_O_3_ : Eu^3+^ nanoparticles (reprinted (adapted) with permission from [48]).

A broad number of dual modal paramagnetic particle systems have been developed [73]. Gd_2_O_3_ nanoparticles for fluorescence/MR imaging have, for example, been reported and doped with Eu^3+^ to produce Gd_2_O_3_ : Eu^3+^ particles. Fluorescence imaging here mediated by Gd^3+^ absorbing a photon and moving to a ^6^*I*_J_ excited state. Energy transfer between this state to the highly unstable ^5^*D*_0_ state of Eu^3+^ is accompanied by the emission of a photon on which the electrons return to the ground state of Eu^3+^. This coupled with a high *r*_1_ = 34.3 mM^−1^·s^−1^ results in an efficient bimodal imaging probe (Figure 2) [48]. CuInS_2_/ZnS quantum dots have also been reported as good candidates for fluorescence/MRI bimodal imaging. These QDs were conjugated with a derivative compound of DTPA for further chelation with Gd^3+^. The composition of these QDs enhanced near-infrared fluorescence (NIRF) and MR imaging with a moderate *r*_1_ = 9.91 mM^−1^·s^−1^ [49]. A recent approach has described the use of carbon dots (CDs) decorated with Gd^3+^ for fluorescence/MRI. In this work, CDs with surface carboxylic groups were obtained via a microwave synthesis. Gd^3+^ was then used to mediate a spherical assembly of the CDs by intercluster electrostatic linkages (-COO^−^-Gd^3+^-^−^OOC). This formulation showed a fluorescence enhancement with increasing Gd^3+^ concentration and a high *r*_1_ value of 32.1 mM^−1^·s^−1^ [50].

A dual MR/CT imaging agent based on surface functionalised bisphosphonate (BP) gadolinium oxide nanoparticles has been developed. Gd_2_O_3_ nanoparticles were synthesised via the polyol method and encapsulated into a mesoporous silica shell by addition of glycidyloxipropyl trimethoxysilane (GPTES). The final formulation presented an *r*_1_ = 15.41 mM^−1^·s^−1^ with *r*_2_/*r*_1_ = 4.77 at 3 T [51].

In addition to contrast agents enabling multiple imaging modalities, dual *T*_1_-*T*_2_ contrast agents can provide enhanced MRI imaging capabilities. Though it is sequence dependent, *T*_2_ agents are, in general, natively disadvantaged since negative contrast generated by the contrast agent can easily be confused with natural artefacts coming from calcification or internal bleeding [74]. One approach providing *T*_1_-*T*_2_ contrast reported by Tirusew et al. used mixed Gd-Dy oxide nanoparticles [52]. Both gadolinium and dysprosium have high magnetic moments with the former promoting a shortening of longitudinal relaxation and dysprosium promoting the transverse relaxation. In an alternative approach, iron oxide nanoparticles were used instead of dysprosium to enhance *T*_2_ contrast. In recent work, for example, Gd-labelled Fe@Fe_3_O_4_ particles have been reported with relaxivity values of *r*_1_ = 7.2 mM^−1^·s^−1^ and *r*_2_ = 109.4 mM^−1^·s^−1^ at 0.5 T [45]. Another example utilises biologically inspired ultrasmall (<10 nm) melanin nanoparticles loaded with Gd-DOTA as a *T*_1_-*T*_2_ dual-modal contrast agent. One of the active components, melanin, is a biological pigment that is paramagnetic in nature and chelates metal ions very strongly. Its use has thus been proposed as a promising alternative to traditional iron oxide or silica-based particles [46].

### 3.2. Manganese

Manganese nanoparticles have been extensively researched as possible *T*_1_ contrast agents with reduced toxicity (compared to that of gadolinium) but however suffer from low native *r*_1_ relaxivities [53, 75]. Much effort has been invested in increasing relaxivity and biocompatibility through the use of derived nanoparticulate systems. For instance, MnO nanoparticles have been encapsulated in polyethylene glycol showing no significant toxicity [16]. More recently, PEG-functionalised Mn_3_O_4_ nanoparticles have been encapsulated in a mesoporous, biocompatible carbon framework, with the latter enabling the water access required while apparently reducing Mn cation loss [17]. Other works conducted by Zhao et al. substituted Fe^2+^ ions on the surface of magnetite particles with Mn^2+^ to produce particles with *T*_1_-weighted imaging capabilities [54]. On increasing the extent of doping of Mn^2+^ on the surface, an element with both a longer electronic relaxation time and greater paramagnetism than Fe^2+^, there was a corresponding increase in *r*_1_.

Hollow Mn_3_O_4_ nanoparticles have been used in a study of the effect of surface functionalisation on relaxometric properties. It was observed, for example, that, with carboxylic acid functionalised ligands, there was a corresponding increase in *r*_1_. This was proposed to be due to the induction of ferromagnetic spins between free surface spins [55]. Coordination polymers (NCP) of nanometric size (78.6 ± 5.4 nm) have been loaded with Mn^2+^ to produce efficient *T*_1_ contrast agents. In this case, organic bridging ligands are used to produce a self-assembly process with Mn^2+^. These nanoparticles displayed high Mn loading of up to 13.3 ± 4 wt.% with a maximum *r*_1_ value of 11.6 mM^−1^·s^−1^. The increase in the relaxivity was assigned to a reduction in the tumbling rates [56].

There has been a growing interest in the development of environmentally responsive MR agents [76]. One major class of these are pH responsive contrast agents which offer potential value in resolving the low pH microenvironment associated with tumour tissue [76]. Manganese-based double-layered hydroxide nanoparticles were the first reported ultrasensitive pH-responsive Mn-based contrast agent for *T*_1_ MRI imaging, showing a 6-fold increase in longitudinal relaxivity values in acidic media (pH = 5) than at pH = 7.4, a switch assigned to the unique structure of Mn ions in the double-layered hydroxide [59]. Another reported pH responsive nanoparticulate example has been the synthesis of Fe_3_O_4_@C@MnO_2_ nanoparticles. Here, iron oxide nanoparticles were coated with a carbon layer and reacted with KMnO_4_ to produce MnO_2_ nanosheets on the outer carbon shells. In a protic environment, these MnO_2_ nanosheets are reduced to Mn^2+^ The increased concentration of paramagnetic Mn^2+^ ions in solution is accompanied by a corresponding increase in *T*_1_ relaxation [60]. Manganese monoxide nanocomposites functionalised with porous gold nanoclusters have also been used as pH-responsive probes. In this work, it was suggested that the gold nanoclusters sterically hinder the release Mn^2+^ from the particles, consequently providing delayed *T*_1_ contrast and a longer diagnostic window. They also allow the system to function as a multimodal probe with photoacoustic and X-ray CT imaging modalities additionally supported [61].

### 3.3. Dysprosium

Dysprosium is one possible alternative to conventional gadolinium-based contrast agents and acts as an effective *T*_2_ contrast agent through its high magnetic moment (the largest of the lanthanides) and short (∼10^−13^ s) electronic relaxation time. Many approaches have been employed to produce dysprosium-modified nanoparticles, most recently MSNs that incorporate Dy-DOTA chelates in the outer pore channel producing particles with high *r*_2_ values of 143.5 ± 8.2 mM^−1^·s^−1^ at 11.7 T, some 20 times larger in magnitude than the molecular analogue [20]. Even higher *T*_2_ relaxivities have been reported by loading Dy-DOTA chelates into the cavity of the tobacco mosaic virus (*r*_2_ values of 326 mM^−1^·s^−1^ at 7 T and 399 mM^−1^·s^−1^ at 9 T) [62]. Dysprosium oxide nanoparticles and dysprosium fluoride have also been proposed as promising agents. For example, González-Mancebo et al. produced DyF_3_ rhombus-shaped nanoparticles with an average size of 110 × 50 nm. These nanoparticles present a remarkable *r*_2_ of 380.4 mM^−1^·s^−1^ when measured at 9.4 T, ascribed to strong outer-sphere effects where the diffusion correlation time (*τ*_*D*_) is affected by the effective radius of the nanoparticles [63]. Dysprosium oxide nanoparticles and dysprosium hydroxide nanorods have also been described as good candidates for *T*_2_ contrast agents. Research by Kattel et al. synthesised d-glucuronic acid-coated ultrasmall Dy_2_O_3_ nanoparticles with an average size of 3.2 nm and Dy(OH)_3_ nanorods with an average size of 20 × 300 nm. The first formulation showed an *r*_2_ = 65.0 mM^−1^·s^−1^ whilst the nanorods possessed a higher value (*r*_2_ = 181.57 mM^−1^·s^−1^ at 1.5 T) due to their greater size [18]. *β*-NaDyF_4_ nanoparticles have shown potential as ultrahigh field magnetic resonance imaging (9.4 T) agents with high *r*_2_ values [19]. Another formulation based on the synthesis of MnCO_3_ nanoparticles doped with Dy exhibited reported *r*_1_ values higher than those typical of MnO NPs [64].

There are numerous reports of dual-modal contrast nanoparticulate agents containing dysprosium. Tb^3+^-doped Dy_2_O_3_ nanoparticles have, for example, been reported to possess both MRI and optical imaging modalities since Tb^3+^ emits across the range of 489 nm to 619 nm, the brightest region being ∼545 nm (green) [65]. PEGylated NaGdF_4_ : Dy nanoprobes as both dual-modal *T*_1_-*T*_2_ and MRI/CT agents were produced by Jin et al. *T*_1_-weighted contrast is provided by the presence of Gd^3+^ ions with *T*_2_-weighted contrast promoted by the doped Dy^3+^ ions, which also facilitates the use of the nanoprobe for CT imaging [66]. Dysprosium has been used to dope Gd_2_O_3_ nanoparticles resulting in dual-modal MR/fluorescence imaging [67]. These particles contain a silica core encapsulated by a Gd_2_O_3_ : Dy^3+^ nanoshell. Altering the thickness of this shell changed the relaxometric properties of the system with the highest *r*_1_ values being reported for the thinnest (2 nm) shells.

### 3.4. Holmium

Holmium is another paramagnetic lanthanide with a highly effective magnetic moment and short electronic relaxation time. The first example of single Ho^3+^-doped upconversion nanoparticles for *T*_2_-weighted MRI was reported by Ni et al., focusing on Ho^3+^-doped NaYbF_4_ with surface phospholipid-PEGylation. Incorporation of both Yb^3+^ as a sensitizer and Ho^3+^ as an activator facilitates upconversion such that optical emission in the visible region is possible. In conjunction with efficient *r*_2_ relaxation, this example is an effective dual-modal contrast agent [77]. NaHoF_4_ nanoparticles have also been shown to be effective for *T*_2_-weighted MRI with reported values of *r*_2_ = 222.6 mM^−1^·s^−1^ at 7 T [68], as have holmium oxide nanoparticles_._ [69] The highest reported *r*_2_ values for holmium, and indeed any of the examples mentioned in this review, are for rhombus-like HoF_3_ nanoparticles produced by González-Mancebo et al. with *r*_2_ = 608.4 at 9.4 T.

**Figure 3:**
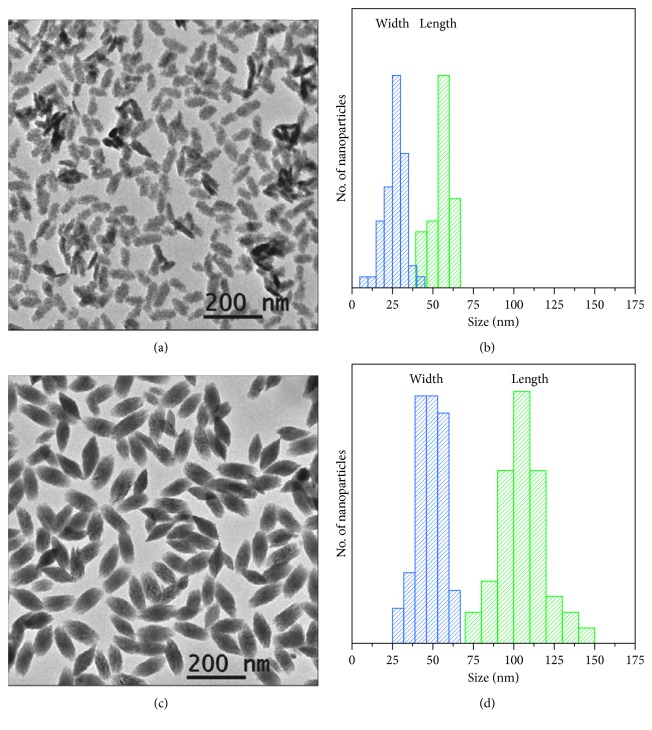
TEM images and size distribution plots obtained from TEM images of (a, b) HoF-el and (c, d) HoF-rh NPs (reprinted (adapted) with permission from [63]).

In this work, two different HoF_3_ formulations were synthesised by homogeneous precipitation in ethylene glycol. Particle size and shape was notably tuneable with Ho(NO_3_)_3_ rendering ellipsoid-like nanoparticles (so-called HoF-el) and 70 × 30 nm in size (Figures 3(a) and 3(b)). An alternative with Ho(CH_3_CO_2_)_3_ as the precursor formed rhombus-like nanoparticles (so-called HoF-rh) with an average size of 110 × 50 nm (Figures 3(c) and 3(d)). Although HoF-el displayed large *r*_2_ value of 350.0 mM^−1^·s^−1^ at high magnetic fields (9.4 T), an unprecedented *r*_2_ value of 608.4 mM^−1^·s^−1^ was reported for HoF-rh indicating not only a size dependency (increased magnetisation of the larger particles) but also pronounced geometric effects [63].

## 4. Multimodal Imaging and Theranostic Applications

Paramagnetic nanoparticles have applications in responsive MRI, targeted imaging, cell tracking, multimodal imaging, and as part of a theranostic platform. Here, some relevant and interesting examples reported across the last few years are described.

Very recently, Gd^3+^ complexes have been integrated into protein, calcium phosphate, polymeric, gold, and bismuth based nanoparticles. These have been utilised as nanotheranostics tools for multimodal imaging and in cancer therapy but also for the chemical imaging of neurotransmitters [78–83]. In 2018, Gd_2_O_3_ nanoparticles were applied to the targeted imaging of integrins for cancer diagnostics, cell labelling studies, and the multimodal imaging of calcium phosphate bone cement [51, 84, 85]. Gd^3+^ has been immobilized into metallofullerenes for MRI and photothermal therapy at tumour sites, within cerium oxide nanoparticles (CeNP) as a promising antioxidant theranostic agent, within leukosomes with enhanced activity towards activated endothelium cells, and on carbon dots for imaging-guided radiotherapy of tumours [40, 86–88].

**Figure 4:**
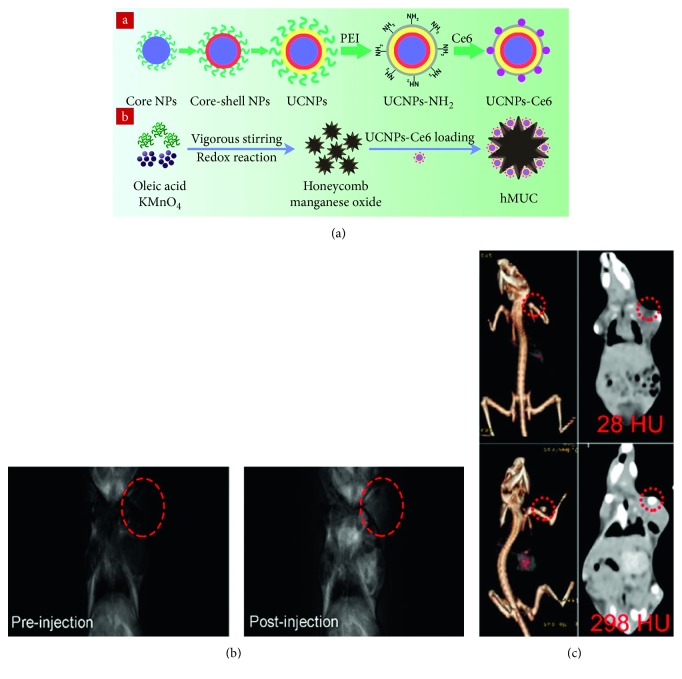
(a) Schematic illustration for the synthesis of UCNPs-Ce6 and hMUC. (b) In vivo *T*_1_-weighted MR images of a tumour-bearing mouse before (left) and after (right) intravenous injection of hMUC. (c) In vivo CT images of a tumour-bearing mouse before (upper) and after (lower) intratumour injection (reprinted (adapted) with permission from [90]).

Liu et al. reported multifunctional redox/pH responsive MnO_2_ nanoparticles for cancer theranostics [89]. Based on honeycomb MnO_2_ nanoparticles (hMnO_2_), Sun et al. have developed pH/H_2_O_2_ responsive nanoparticles loaded with the photosensitiser chlorin e6 (Ce6). These upconverting nanoparticles, denoted as hMUC (Figure 4(a)), support high in vivo MRI *T*_1_ contrast within tumours (Figure 4(b)). The presence of high Z-elements also facilitated CT imaging (Figure 4(c)). Finally, the particles were able to produce reactive oxygen species (ROS) through action of the Ce6 photosensitiser enabling tumour treatment by photodynamic therapy (PDT) [90].

Similar methodologies have been reported in enabling the pH and/or redox responsive diagnosis and photodynamic therapy of tumours using MnO_2_ nanoparticles coupled with gold nanocages, copper sulfide nanostructures, or iron oxide nanoparticles [91–94].

Wang et al. have recently reported the synthesis of holmium-doped hollow silica nanospheres to create multifunctional theranostic nanoparticles. These were subsequently conjugated with a prostate stem cell antigen (PSCA) monoclonal antibody for targeted bimodal US/MRI of tumours as well as combined sonodynamic and hypoxia activated therapy [95]. Another application of multifunctional nanoparticles for dual-modal imaging is reported by Li et al., where ternary-doped (fluorine, ytterbium, and holmium) hydroxyapatite nanoparticles were used for multimodal imaging/tracking of hydroxyapatite in hard tissue repair [96]. Pegylated NaHoF_4_ nanoparticles have also been employed for single MR and MR/CT dual-modality imaging applications [68, 97].

**Figure 5:**
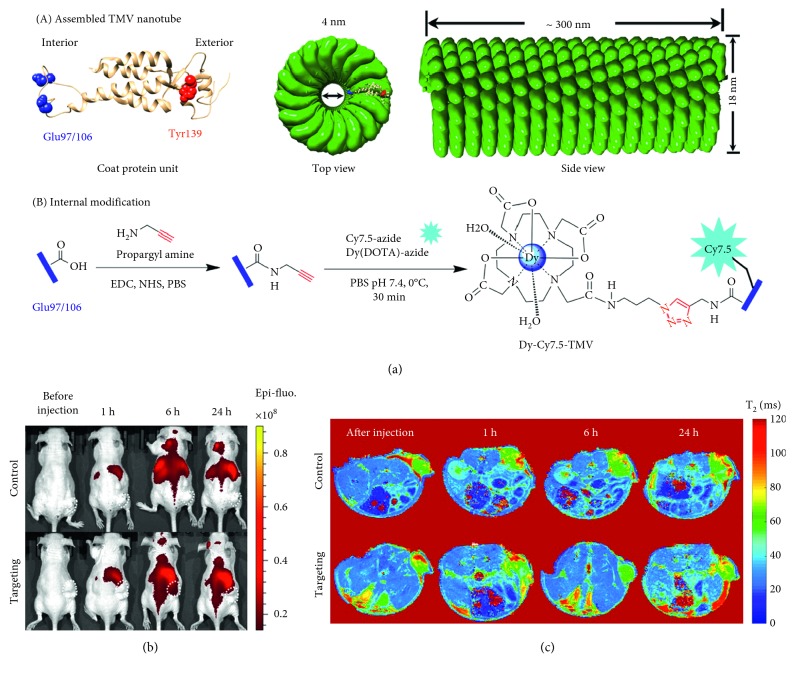
(a) Structure of the tobacco mosaic virus (TMV) nanoparticle's coat protein with surface-exposed residues highlighted as internal glutamic acid (blue) and external tyrosine (red) and the structure of the assembled capsid/strategy for internal modification. (b) Near-infrared fluorescence (NIRF) imaging of subcutaneous PC-3 (*α*2*β*1) prostate tumours in athymic nude mice (*n* = 3) before and 1, 6, and 24 h after the intravenous injection of Dy-Cy7.5-TMV-mPEG (control group) or Dy-Cy7.5-TMV-DGEA (targeting group). (c) In vivo *T*_2_-mapping MRI of subcutaneous PC-3 (*α*2*β*1) prostate tumours in athymic nude mice (*n* = 3) obtained before and 1, 6, and 24 h after the intravenous injection of Dy-Cy7.5-TMV-mPEG (control group) and Dy-Cy7.5-TMV-DGEA (targeting group) (reprinted (adapted) with permission from [62]).

A notable example is reported by Hu et al. where dysprosium-modified tobacco mosaic virus nanoparticles were used for the bimodal MR/NIRF imaging of prostate cancer. In this work, internal glutamic acid residues were exploited to functionalise the nanoparticles with Dy-DOTA-azide and a NIRF dye Cy7.5-azide by a copper-catalysed azide-alkyne click chemistry cycloaddition (Figure 5(a)). Then, the external surface was conjugated with a DGEA peptide that binds to a specific integrin on the surface of PC-3 prostate cancer cells. The final formulation was able to produce targeted NIRF imaging in nude mice (Figure 5(b)), displaying strong *T*_2_ contrast as supported by a high transverse relaxivity (399 mM^−1^·s^−1^ at 9.4 T) (Figure 5(c)) [62].

## 5. Conclusions

Paramagnetic contrast agents present themselves as valuable tools for a broad range of potent MRI applications. Although traditional paramagnetic coordination complexes have been extensively applied, control over both thermodynamic and kinetic stability, pharmacokinetics, biodistribution, and imaging potency is limited. There are a wide variety of accessible synthetic procedures to develop nanoparticles conjugated with paramagnetic ions with a control over composition, size, and shape. This supports facile management of stability, pharmacokinetics, and biodistribution. Associated paramagnet hydration, water exchange kinetics, or residence lifetime can be tuned, all at high levels of local loading. The ability to readily integrate additional imaging modes and the employment of multivalent vectors across comparatively high particle surface areas make these yet more promising. To date, these constructs have been applied to the targeted multimodal molecular imaging, of cancer, cardiovascular, and neurological diseases as well as drug delivery, photodynamic, and sonodynamic therapy. One would fully expect the chemical richness available here to be truly impactful over the next decade.

## Figures and Tables

**Table 1:** Examples of nanoparticle-based paramagnetic contrast agents for magnetic resonance imaging included in this review.

Material	Size (nm)	*r*_1_ (mM^−1^·s^−1^)	*r*_2_ (mM^−1^·s^−1^)	Magnetic field (*T*)	Reference
Gd-Si-DTTA	37	19.7	60.0	3	[37]
MSN-DTTA-Gd	75	28.8	65.5	3	[38]
Gd-DOTA-MSNs	66.3 ± 6.6	33.6 ± 1.3	—	7	[39]
CeOx : Gd9%	3.8	13.4	25.8	1.41	[40]
GO-DOTA-Gd	20–50	14.2	—	11.7	[41]
iGd-TMV-Si	300 × 18 nm, 4 nm internal channel	29.7	—	1.41	[42]
Gd-DND	4.9	33.4	332.0	8	[43]
Hsp DTPA-Gd nanocage	37.1	47.4	—	1.5	[44]
DOTA(Gd)-Fe@Fe_3_O_4_	258	7.2	109.4	0.5	[45]
Gd-M-dots	14.58	23.4	123.3	3	[46]
Gd_2_O_3_	1.0	9.9	10.5	1.5	[15]
Silica-Gd_2_O_3_	91.5	30.8	—	0.55	[47]
Gd_2_O_3_ : Eu^3+^	7.4 ± 0.3	34.3	—	3	[48]
QDs@DTDTPA-Gd	24.7 ± 2.7	9.9	—	3	[49]
A-C-dots@Ce6	98 ± 10	32.1	—	3	[50]
GBCAs-BP	70	15.4	73.5	3	[51]
GDO(Gd + Dy)	1.0	6.0	40.0	1.5	[52]
PEG-MnO	1.9	12.9	60.3	3	[53]
MnO	7	0.4	1.7	3	[16]
Mn_3_O_4_@CF	~70	3.5	—	1.41	[17]
MnIO	17.3	57.8 ± 6.5	306.3 ± 15.2	0.5	[54]
OA-PL-HMON	—	1.1	9.2	3	[55]
NCP@peg-AA	78.6 ± 5.4	11.6	19.7	3	[56]
NPs-dopa-PEG-DOTA/RGD	26.4 ± 7.5	—	267.5	7	[57]
^64^Cu-NOTA-FA-FI-PEG-PEI-Ac-Mn_3_O_4_	476.5 ± 13.5	1.0	—	0.5	[58]
Mn-LDH	48.0 ± 1.8	9.5 at pH 5.0 1.2 at pH 7.4	—	16.4	[59]
Fe_3_O_4_@C@MnO_2_	150	5.3 at pH 5 2.2 at pH 7.4	364.2 at pH 5 442.4 at pH 7.4	3	[60]
MnO@Au NCs	45.0 ± 5.1	2.4 at pH 5.4 1.2 at pH 7.4	—	7	[61]
Dy-MSNs-L	166.2 ± 1.9	—	143.5 ± 8.2	11.7	[20]
Dy(DOTA)-Cy7.5-TMV-PEG-DGEA	300 × 18 nm, 4 nm internal channel	—	399.0	9.4	[62]
DyF_3_	100.35	0.9	380.4	9.4	[63]
D_2_O_3_ (D-glucuronic acid coating)	3.2	0.008	65.0	1.5	[18]
NaDyF_4_	20.3	0.3	101.0	9.4	[19]
Dy-doped MnCO_3_	9.27 ± 0.72	4.5	—	7	[64]
Dy_2_O_3_ : Tb^3+^	3.0 ± 0.3	—	2.2	7	[65]
PEG-NaGdF_4_:Dy	—	5.2	10.6	9.4	[66]
SiO_2_@Gd_2_O_3_ : Dy^3+^	101.5	30.2	—	0.55	[67]
NaHoF_4_	28.9	0.6	222.6	7	[68]
PEG-Ho_2_O_3_	80–90	—	23.5	1.5	[69]
HoF_3_	94.3	0.6	608.4	9.4	[63]
